# A patient-derived orthotopic xenograft model enabling human high-grade urothelial cell carcinoma of the bladder tumor implantation, growth, angiogenesis, and metastasis

**DOI:** 10.18632/oncotarget.26024

**Published:** 2018-08-24

**Authors:** Jessie Gills, Ravan Moret, Xin Zhang, John Nelson, Grace Maresh, Linh Hellmers, Daniel Canter, M’Liss Hudson, Shams Halat, Marc Matrana, Michael P. Marino, Jakob Reiser, Maureen Shuh, Eric Laborde, Maria Latsis, Sunil Talwar, Stephen Bardot, Li Li

**Affiliations:** ^1^ Department of Urology, Ochsner Clinic Foundation, New Orleans, LA, USA; ^2^ Institution of Translational Research, Ochsner Clinic Foundation, New Orleans, LA, USA; ^3^ Department of Pathology, Ochsner Clinic Foundation, New Orleans, LA, USA; ^4^ Department of Hematology and Oncology, Ochsner Clinic Foundation, New Orleans, LA, USA; ^5^ Division of Cellular and Gene Therapies, The Center for Biologics Evaluation and Research, US Food and Drug Administration, Silver Spring, MD, USA; ^6^ Current address: Memorial Urology Associates, Houston, TX, USA

**Keywords:** patient-derived orthotopic xenograft, high-grade/muscle invasive urothelial cell carcinoma, lymph node stromal cells

## Abstract

High-grade urothelial cell carcinoma of the bladder has a poor prognosis when lymph nodes are involved. Despite curative therapy for clinically-localized disease, over half of the muscle-invasive urothelial cell carcinoma patients will develop metastases and die within 5 years. There are currently no described xenograft models that consistently mimic urothelial cell carcinoma metastasis. To develop a patient-derived orthotopic xenograft model to mimic clinical urothelial cell carcinoma progression to metastatic disease, the urothelial cell carcinoma cell line UM-UC-3 and two urothelial cell carcinoma patient specimens were doubly tagged with Luciferase/RFP and were intra-vesically (IB) instilled into NOD/SCID mice with or without lymph node stromal cells (HK cells). Mice were monitored weekly with bioluminescence imaging to assess tumor growth and metastasis. Primary tumors and organs were harvested for bioluminescence imaging, weight, and formalin-fixed for hematoxylin and eosin and immunohistochemistry staining. In this patient-derived orthotopic xenograft model, xenograft tumors showed better implantation rates than currently reported using other models. Xenograft tumors histologically resembled pre-implanted primary specimens from patients, presenting muscle-invasive growth patterns. In the presence of HK cells, tumor formation, tumor angiogenesis, and distant organ metastasis were significantly enhanced in both UM-UC-3 cells and patient-derived specimens. Thus, we established a unique, reproducible patient-derived orthotopic xenograft model using human high-grade urothelial cell carcinoma cells and lymph node stromal cells. It allows for investigating the mechanism involved in tumor formation and metastasis, and therefore it is useful for future testing the optimal sequence of conventional drugs or the efficacy of novel therapeutic drugs.

## INTRODUCTION

In the United States in 2018, there will be an estimated 81,190 new patients and 17,240 cancer specific deaths attributable to urothelial cell carcinoma (UCC) of the bladder [1, 2]. Although the majority (70%) of patients will present with non-muscle invasive disease, the remaining 30% of patients will have muscle invasive disease (MIUCC) [3]. 50% of patients with MIUCC of the bladder, despite attempted curative therapy, e.g., radical cystectomy (RC) with or without systemic chemotherapy, for clinically localized disease, will still develop metastases and die within 5 years [1]. Approximately 20–25% of patients undergoing RC are found to have nodal metastasis [4–6]. Five-year survival after RC alone for node positive disease is less than 35% at best; making nodal involvement an important negative predictor of patient survival. Adjuvant chemotherapy for lymph node (LN) involvement after RC has conflicting efficacy reports [7–11]. Apart from adjuvant chemotherapy, there exist few therapeutic options in the advanced and/or metastatic setting, creating a need for novel therapies with better patient-specific treatment options. More recently, clinical trials of immunotherapies in UCC of the bladder have become available for selected patients [12]. However, immunotherapies are limited by a low response rate and the challenge of targeted patient selection [13].

Developing a patient-derived orthotopic xenograft model (PDOX) provides an important platform for translational cancer research [14, 15]. PDOX models mostly retain the principal histological and genetic characteristics of their donor tumor and remain stable across passages [15, 16]. These models can be predictive of clinical outcomes and are being used for preclinical drug evaluation, biomarker identification, biologic studies, and preclinical evaluation of personalized medicine strategies. Since lymphovascular invasion carries one of the highest risks for overt or occult metastasis [2, 17–23], the interaction between UCC and LN stromal cells (LNSCs) can be crucial for the outcome of UCC. However, at present, there are no described UCC xenograft models that consider LN involvement and consistently reproduce primary tumor in bladder and distant organ metastasis in UCC. In this report, we describe the development of a novel PDOX model in NOD/SCID mice that reproduces metastatic UCC under the influence of LNSCs.

## RESULTS

### Establishing a novel MIUCC PDOX mouse model

**Figure 1:**
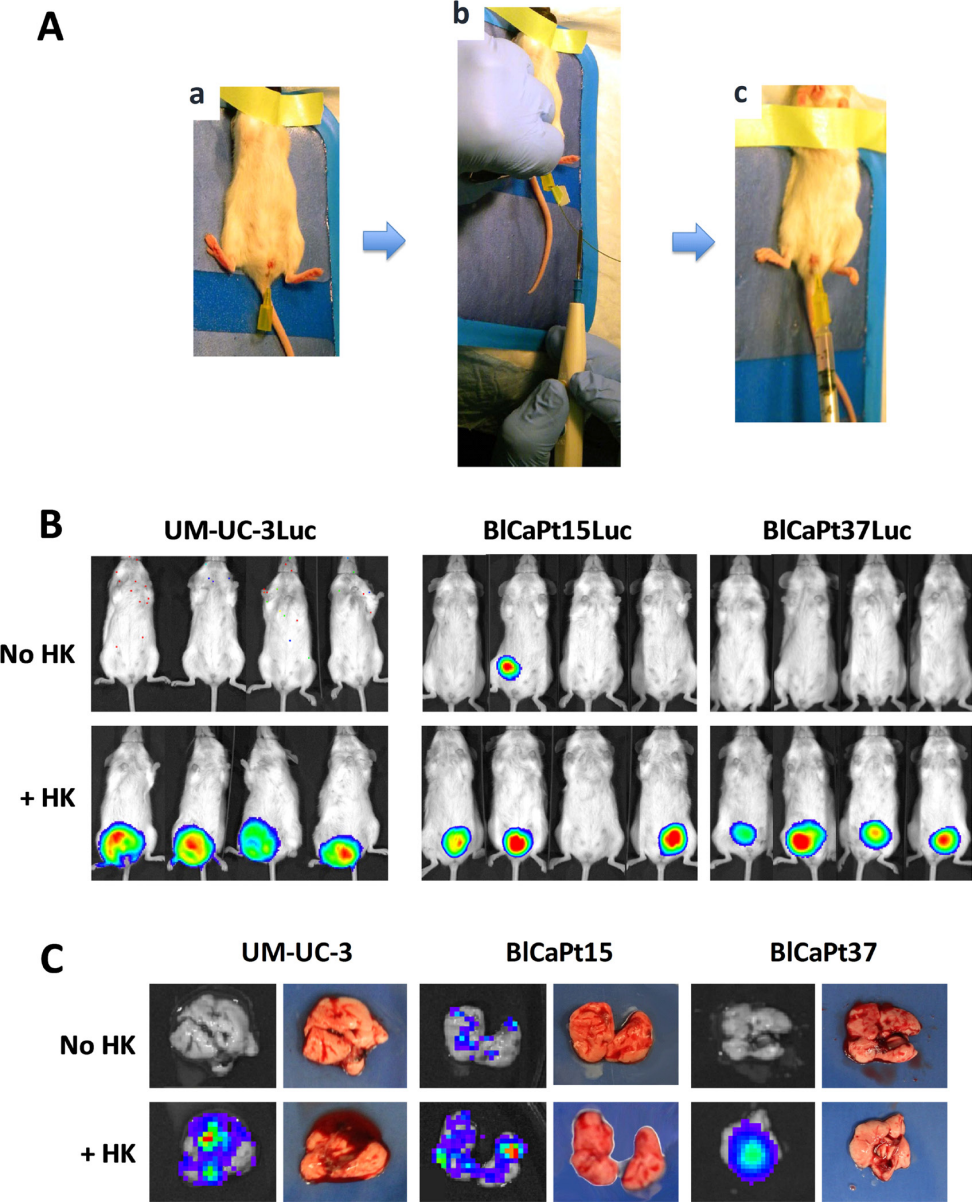
Establishment of an intra-vesicle (IB) instillation based human UCC mouse model (**A**) An angiocatheter was inserted into the bladder of female NOD/SCID mice (**a**); An electrocautery shock was applied to the bladder wall via a guide wire (**b**); and luciferase tagged UCC tumor cells, UM-UC-3-Luc (1 × 10^3^ cells), BlCaPt15-Luc (2 × 10^4^ cells), or BlCaPt37-Luc (5 × 10^5^ cells) with and without the addition of 3 × 10^5^ LN stromal HK cells, were instilled into NOD/SCID mouse bladder through the angiocatheter (**c**). (**B**) Tumor burden was monitored and quantified via bioluminescent imaging (BLI). (**C**) *Ex vivo* BLI was performed for harvested lungs at the endpoint to quantify distant organ metastasis. Group size is as indicated in Table 1. Representative mice or lung images are shown.

Using the UCC cell line UM-UC-3, UCC patient specimens, and intra-vesical (IB) instillation (Figure 1A), we were able to create a novel PDOX IB model in NOD/SCID mice for MIUCC. Tumor formation in the bladder was consistent and reproducible (Figure 1B), especially in the presence of LN stromal HK cells. We did not observe tumor cells spreading to organs near the bladder due to manipulation at the time of IB tumor cell implantation in the mice.

To monitor for extra-nodal metastases in the model, mice lungs were collected at necropsy for *ex vivo* bioluminescence imaging (BLI). As shown in Figure 1C, some mice had Luc^+^ cancer cells present in their lungs. This occurrence was more prevalent when tumor cells were co-inoculated with HK cells. Table 1 summarizes tumor formation and lung metastasis incidence for the UM-UC-3 cell line and patients’ tumor cells BlCaPt15 and BlCaPt37. UM-UC-3 cells produced tumors in 100% of the animals when HK cells were added. Depending on the individual patient tumor type, there was a 59–89% tumor incidence with approximately 50% of the mice having metastases in the presence of HK cells.

**Table 1:** Summary of tumor formation and distant organ metastasis in IB model

	UM-UC-3		BlCaPt15		BlCaPt37	
	Tumor formation	Metastasis	Tumor formation	Metastasis	Tumor formation	Metastasis
**No HK**	0/6 (0%)^a^	0/6 (0%)	4/15 (27%)	2/13 (15%)^b^	0/18 (0%)	0/18 (0%)
**+ HK**	5/5 (100%)	5/5 (100%)	16/18 (89%)	10/18 (56%)	10/17 (59%)	8/17 (47%)
***P* value**	0.0009^c^	0.0009	0.0003	0.0235	0.0001	0.0009

^a^: data show number of mice with tumor formation or metastasis/number of mice tested (%), based on BLI analysis.

^b^: two mice died prior to sacrifice, thus there are no *ex vivo* lung BLI data.

^c^: χ^2^ tests.

### LN stromal cells support human UCC tumor development in the context of the newly developed PDOX mouse model

**Figure 2:**
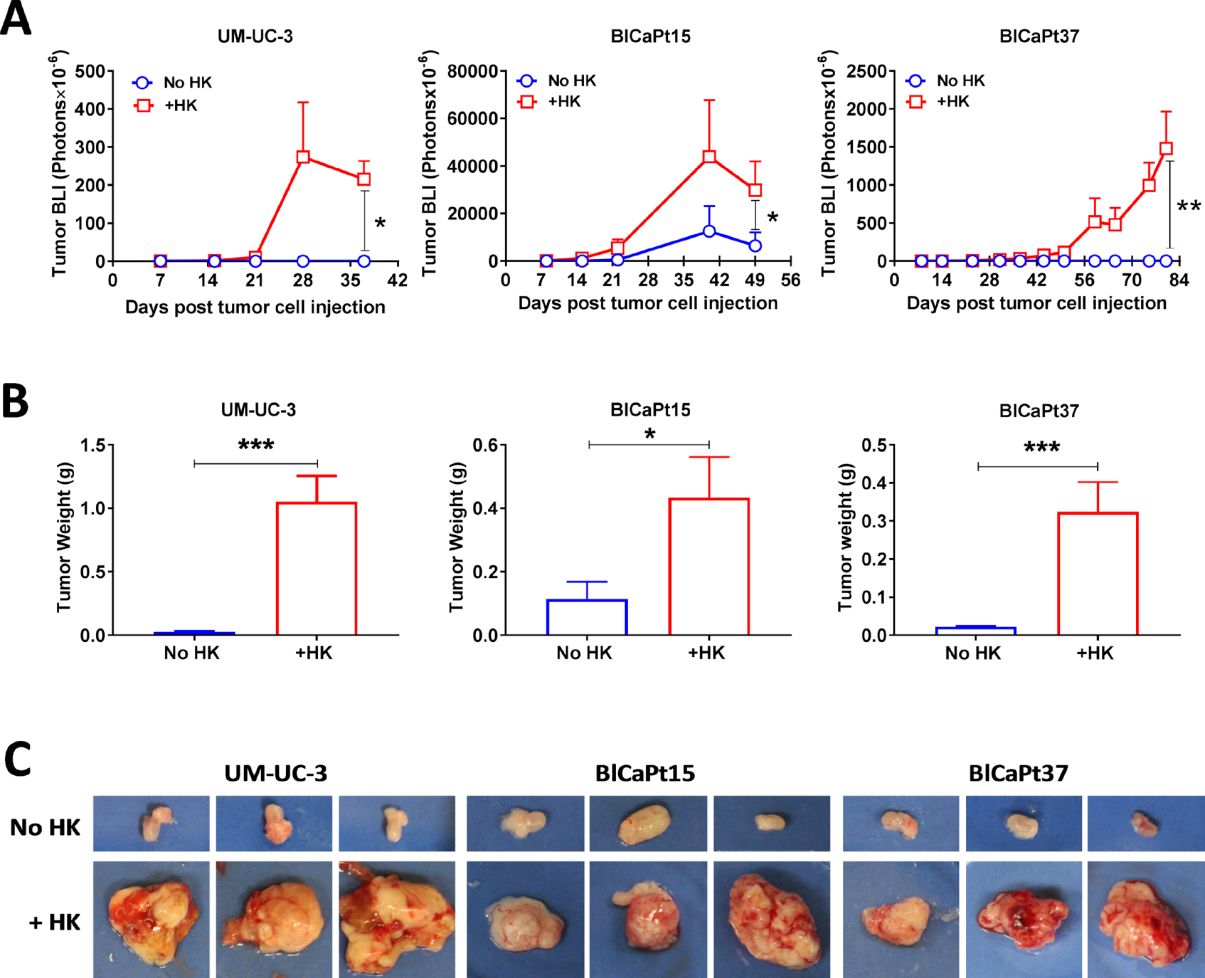
LN stromal HK cells stimulate UCC tumor growth in the IB model (**A**) Tumor growth involving luciferase tagged UCC cells (same experiments as in Figure 1) was monitored kinetically via BLI and analyzed using Living Imaging software. (**B** and **C**) To confirm the BLI findings, bladders with or without tumors were removed from mice upon necropsy, photographed (representatives shown in **C**), and weighed (**B**). Student *t*-test analysis (Prism GraphPad software) was used to calculate statistical significance between groups with or without LN stromal HK cells in tumor BLI (**A**) and tumor weight (**B**). *P <* 0.05 was considered statistically significant, asterisks represent significance: ^*^P < 0.05; ^**^P < 0.01; and ^***^P < 0.001.

Previously, we reported that LNSCs promoted primary tumor formation of colon cancer and B cell lymphoma in orthotopic mouse models [16, 24]. To evaluate the role of LNSCs in UCC tumor growth, varying doses of UCC cells were co-instilled IB with or without HK cells to NOD/SCID mice for tumor formation in our PDOX model. Weekly BLI was performed to monitor tumor growth. When UM-UC-3-Luc cells were used, tumors formed in mice bladders only in the presence of HK cells (Figures 1 and 2 and Table 1).

Patients’ BlCaPt15 and BlCaPt37 cells displayed tumor growth curves and HK cell dependency similar to those of the UM-UC-3-Luc cell line, generating primary tumors in the presence of HK cells in our unique PDOX IB model. Prior to animal sacrifice, final BLI imaging was performed. Figure 2 graphically displays that the mixture of UCC and HK cells significantly promoted tumor formation. Although tumors formed in 27% of the mice that were injected with tumor cells alone, 89% of mice instilled with BlCaPt15 tumor cells in the presence of HK cells formed tumors (Figure 2 and Table 1). Also, mice which had BlCaPt15 tumor cells co-inoculated with HK cells developed tumors with a shorter latency period and a faster growth rate when compared to mice that received cancer cells alone (Figure 2A). In the presence of HK cells, mice with as few as 1 × 10^3^ UM-UC-3 cells or 2 × 10^4^ BlCaPt15 tumor cells formed xenograft tumors in 3 weeks, while mice with 5 × 10^5^ BlCaPt37 tumor cells formed xenograft tumors in 7 weeks post cancer cell injection (Figure 2A). Mice injected with BlCaPt37 tumor cells alone did not form tumors, but when co-injected with HK cells, 59% of mice formed tumors in the bladder (*P* = 0.00989, Figure 2A and Table 1). Of note, histologically, BlCaPt15 is a high-grade UCC with squamous differentiation, a more aggressive type of tumor compared with BlCaPt37, which is a high-grade UCC without squamous differentiation (Table 2) [25]. This difference was recapitulated in our model system where the former requires fewer tumor cells and a shorter latency period for tumor formation.

**Table 2:** Patient demography and pathology diagnoses

	Age (sex)	Pathology stage	Histological grade	Histological type	Tumor size
**BlCaPt15**	59 (F)	pT3b N1 M0	High grade	Urothelial (transitional cell) carcinoma with squamous differentiation	9 × 5.5 cm
**BlCaPt37**	68 (M)	pT3b pN0 M0	High grade	Urothelial carcinoma	4 × 2 cm

In the presence of HK cells, the UM-UC-3-Luc cells, BlCaPt37-Luc cells, and BlCaPt15-Luc cells demonstrated statistically significant higher tumor weights when compared to in the absence of HK cells (*P* = 0.0003, *P* = 0.04, and *P* = 0.0004, respectively, Figure 2B). Taken together, mice given bladder cancer cells with the addition of HK cells developed statistically larger tumors when compared with mice receiving cancer cells alone (Figure 2B and 2C).

### LN stromal cells augment human UCC tumor angiogenesis in the unique PDOX mouse model

**Figure 3:**
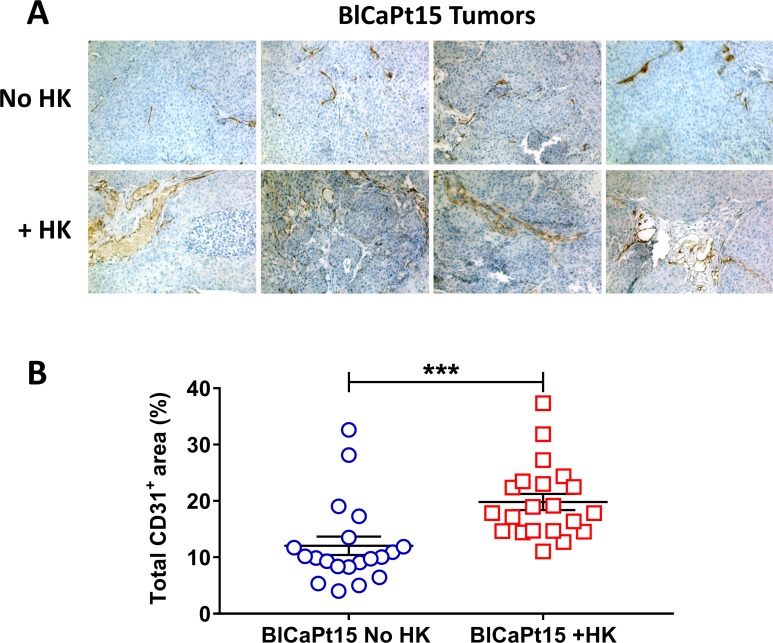
LN stromal HK cells support UCC tumor angiogenesis in the IB model Xenograft tumors from mice inoculated with BlCaPt15 cells with or without LN stromal HK cells (same experiments as in Figure 1) were collected upon necropsy. Tumors were fixed, embedded, sectioned at 5μm and stained by IHC using antibody against mouse CD31 to detect angiogenesis (brown staining). (**A**) Photographs were taken using a digital deconvoluting microscope and Slide Book 6.0 software. Original magnification 200×. Representative images are shown. (**B**) Captured images were uploaded to Aperio ImageScope software (Aperio Technologies in Vista, Ca) for digital analyses for the percentage positive (brown) area on each photograph. The difference between mice inoculated with BlCaPt15 cells with or without LN stroma HK cells was analyzed using Student *t*-test, *P* = 0.0009.

Tumor angiogenesis is a hallmark of tumor progression [26]. To reveal the mechanism of UCC progression and host angiogenesis in the presence of HK cells, an antibody against the mouse endothelial cell marker Cluster of Differentiation 31 (CD31) was used for immunohistochemical (IHC) staining. CD31 is expressed constitutively on the surface of endothelial cells and is involved in the mediation of cell-to-cell adhesion. CD31-mediated endothelial cell-cell interactions play a major role in angiogenesis. In our model, tumor microvasculature was detected by CD31 staining in tumors formed by BlCaPt15 tumor cells using IHC. Comparing CD31-positive staining between tumors formed by instilling tumor cells alone and tumor cells plus HK cells, BlCaPt15 cell primary tumor angiogenesis was significantly enhanced in the presence of HK cells as indicated by digital analysis of the percentages of vasculature covered tumor areas, (Figure 3, dark brown staining, *P* = 0.0009). The rate of tumor formation incidence measured by BLI also confirmed the LN stromal cell support with statistical significance (Table 1). Thus, the presence of HK cells supports UCC tumorigenesis at least partly through promoting host vascular development or angiogenesis. Due to the fact that tumor formation was not observed with UM-UC-3 cells and BlCaPt37 tumor cells in the absence of HK cells, similar comparison tests were not performed.

### LN stromal cells support human UCC extra-nodal metastasis in the novel PDOX mouse model

**Figure 4:**
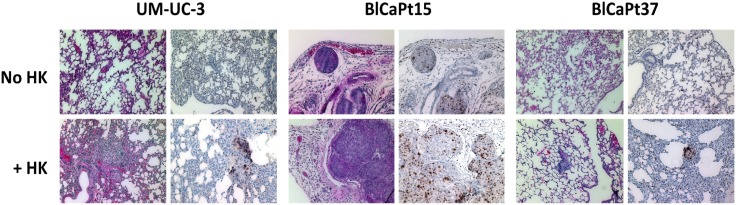
LN stromal HK cells promote UCC tumor lung metastasis in the IB model Paraffin imbedded lungs collected from representative mice injected with UM-UC-3, BlCaPt15, or BlCaPt37 tumor cells with or without HK cells in the IB model (same experiments as in Figure 1) were sectioned and stained by H&E or IHC with antibodies against human Ki67. Photographs were taken using a digital deconvoluting microscope and Slide Book 6.0 software. The brown color indicates positive staining. Original magnification 200×.

Similar to local tumor formation, when investigated by *ex vivo* BLI of the lungs, no metastatic disease was observed in mice injected with cancer cells alone, however when mice were co-inoculated with HK cells, lung metastases developed in all mice injected with UM-UC-3 cells and 47% of mice injected with BlCaPt37 tumor cells (Table 1). In the case of BlCaPt15 tumor cells, which are derived from a more aggressive tumor with confirmed LN metastasis (Table 2), 15% and 56% mice developed lung metastases when tumor cells were injected alone or in the presence of HK cells, respectively. In addition, lung metastasis detected by *ex vivo* BLI were confirmed using IHC analysis. When the tumor bearing mouse lungs were tested by hematoxylin and eosin (H&E) staining and IHC of the human cell proliferation marker Ki67, staining revealed Ki67^+^ human tumor cells in the lungs of mice injected with all 3 different tumor cells when they were co-inoculated with HK cells (Figure 4). Therefore, our PDOX model recapitulated UCC patient clinical progression. These results underscore the crucial role that the LN stromal microenvironment plays in UCC pathogenesis.

### The PDOX mouse model recapitulates MIUCC in patients

**Figure 5:**
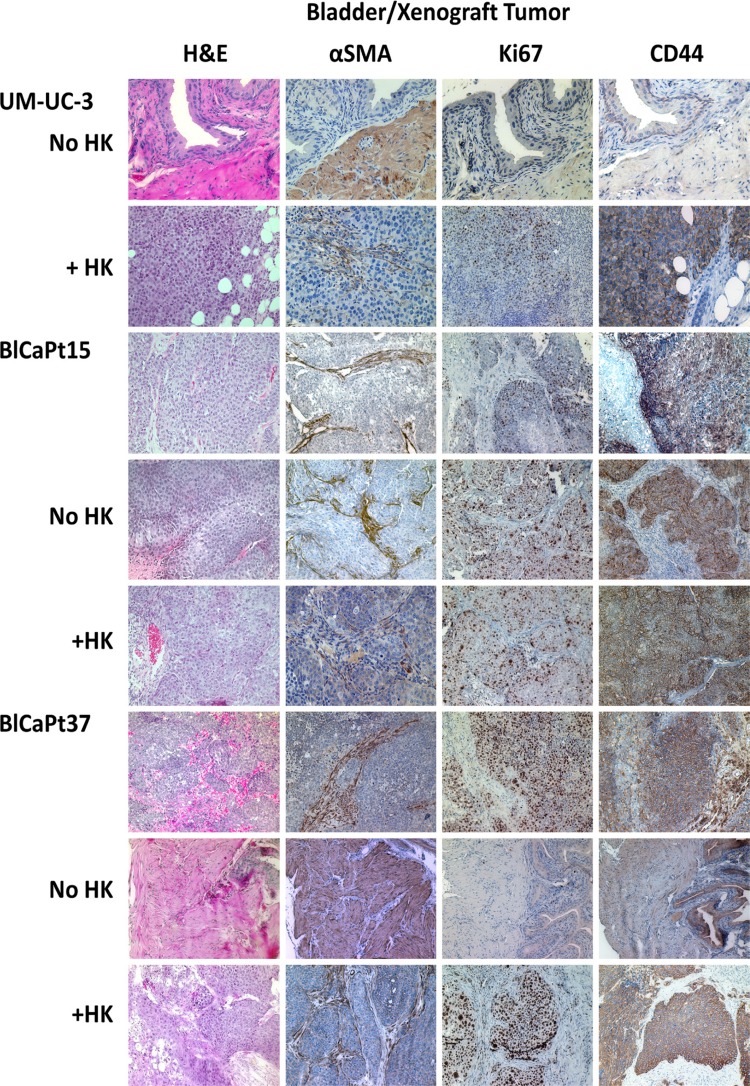
The IB model recapitulated the architectural and molecular expression characteristics of UCC patient primary tumors Primary UCC patient bladder tumor specimens (BlCaPt15 and BlCaPt37), and bladder/tumor from their corresponding xenografts and UM-UC-3 cell xenografts in the IB model (same experiments as in Figure 1) were sectioned and stained by H&E or IHC with antibodies against mouse and human αSMA, human Ki67, or human CD44. H&E stain shows tumor nests dissecting into smooth muscle bundles. SMA stain highlights these muscle bundles and shows tumor cells dissecting through them. Photographs were taken using a digital deconvoluting microscope and Slide Book 6.0 software. The brown color indicates positive staining. Original magnification 200×.

To confirm architectural similarity, H&E and IHC staining was performed on xenografts and primary patient tumors. Histopathology of the xenografts resembled patient bladder carcinoma in both cases of BlCaPt15 and BlCaPt37. The antibody specific to both human and mouse alpha-smooth muscle actin (α-SMA) was used. The positive staining indicates that in both patient xenografts, tumor growth penetrated the basement membrane and lamina propria and invaded into the muscle layer, corresponding to the growth pattern of the patients’ primary tumors (Figure 5). In addition, the UCC cell line UM-UC-3 xenografts also showed a muscle invasive growth pattern in mouse bladder when cancer cells were co-inoculated with LN stromal HK cells.

CD44^+^ membrane staining and Ki67^+^ nuclear staining indicate highly proliferative, fast-growing human tumor cells. Tumors formed in mouse bladder from human UM-UC-3-Luc, BlCaPt15-Luc, and BlCaPt37-Luc cells, especially in the presence of HK cells, were detected by CD44 and Ki67 IHC staining. The staining pattern of tumors formed in mice from patient specimens were comparable to those of the original surgical biopsies (Figure 5). Thus, in our model, xenograft tumors resembled pre-implantation tumor specimens from UCC patients, demonstrating a muscle invasive growth pattern.

## DISCUSSION

In this report, we describe a novel orthotopic metastatic human UCC xenograft model in which IB instilled UCC cells from a cultured cell line or patient tumor specimens form tumors in the bladder of NOD/SCID mice and metastasize to the lung especially in the presence of LNSCs, which resembles the clinical progression seen in human MIUCC. In addition to tumor implantation, tumor angiogenesis, muscle invasion, and distant organ metastasis were significantly greater in mice with the addition of LNSCs. Of note, tumor morphology and molecular expression characteristics of the PDOX primary tumor were confirmed to match that of the original patient tumors.

Murine models using cancer cells derived from patient tumors (xenografts) allow for a better understanding of tumor biology and predictive biomarkers as well as testing of antineoplastic effects of novel therapies [27]. They have been used to study cancer biology and response to treatment. There have been multiple forms of xenograft models for UCC beginning in the 1970s, including tumor implantation via open cystotomy or trans-abdominal intramural injection, syngeneic and transgenic murine models, and dietary administration of tumor promoting chemicals [27–30]. IB inoculations have been used to create non-muscle invasive bladder tumors, but they were cited as unreliable in tumor engraftment and unpredictable in tumor location. Direct injection of tumor cells into the bladder wall leads to the formation of intramural bladder tumors that are suitable for systemic treatments, but this is a time consuming and invasive procedure, and tumors implanted do not follow the natural course of the disease [27, 30]. The technique of IB instillation with the addition of electro-stimulation with a wire was developed earlier in different mouse models [31–33]. We modified and adjusted the technique to be suitable for our PDOX mouse model.

This is the first time a PDOX for UCC with human LNSCs demonstrating consistent invasive and metastatic disease is described. Our method of IB electro-stimulation with the addition of HK cells produces a reliable model for developing MIUCC, and recreates the natural disease course of UCC by beginning with tumor implantation in the mucosa, followed by invasion into the muscle, then metastasis to lungs. Although our tumor implantation rates, especially with patient tumor cells, may not be as high as direct injection, our model offers several advantages: the method is free from the morbidity that comes with the major surgery needed to expose the bladder; is technically straightforward; allows for the inoculation of a large number of mice in a short time; and the addition of HK cells shortened the experimental period which is very important for chemotherapeutic drug screening.

Dual labeling of UCC cells with Luc/RFP enabled us to 1) enrich for tagged tumor cells without altering tumor cell biology by adding selective antibiotics and 2) monitor tumor progression including primary tumor growth kinetics and track distant organ metastasis. In our unique PDOX model, we used BLI which allows for real time evaluation of tumor growth and metastasis kinetically. BLI has been validated as an accurate method for monitoring tumor growth in numerous xenograft models and allows for non-invasive evaluation of tumor progression and testing responses to targeted therapies [16, 30]. The increased activities seen in BLI in our PDOX model reflect increased tumor burden. A small drawback of our model is the time needed to efficiently tag patient-derived tumors with Luc/RFP in order to track tumor progression by BLI. The availability of PET/CT for small animals may overcome these problems and benefit patients in a timely manner.

Interestingly, in this model, the dependency on LNSCs for tumor formation and metastasis depends on the tumor's aggressiveness although only two patient specimens were tested. Using a high-grade stage III UCC with squamous differentiation and LN involvement tumor, the cells formed tumors and developed metastases although a lower percentage of mice tested positive compared to mice receiving tumor cells co-injected with HK cells. However, a stage III UCC but without squamous differentiation or less aggressive tumor was more dependent on HK cells and needed more tumor cells to initiate tumor growth in the bladder. It also took longer to form tumors and metastasized to lungs in a lower percentage of mice. Urothelial carcinoma with squamous differentiation tends to be more aggressive than those without squamous differentiation [25]. Both patients from whom the cell lines were derived presented *de novo* with muscle invasive disease and underwent three cycles of neoadjuvant systemic chemotherapy (gemcitabine/cisplatin) prior to RC. Neither of these patients had a history of non-muscle invasive bladder cancer for which they received prior to intravesical therapy. One of the weaknesses of this study is the limited number of patients with narrow disease stage who were used, however our findings make testing of additional patient specimens from various disease stages advisable.

In conclusion, our unique orthotopic PDOX model provides a reliable, reproducible platform in which metastatic UCC under the influence of LNSCs can be studied effectively. This model will permit screening of conventional and/or novel therapeutic drugs that target tumor formation and distant organ metastasis within a reasonable time frame that does not diminish its clinical usefulness. Historically, implantation of tumor cells into immune compromised mice has limited the ability to study immune targeted therapies [28]. One of our future directions is to reconstitute a human immune system in this mouse model, then use the humanized mice to study immunotherapy options for individual patients making personalized therapeutic strategies possible for UCC patients. We anticipate that this unique model will be very useful to study the mechanism(s) of the progression of UCC as well as the translational development of novel therapies and selection of treatment strategies for aggressive bladder cancer.

## MATERIALS AND METHODS

### Cell lines and cell culture

The human bladder cancer cell line UM-UC-3 was purchased from the American Type Culture Collection (ATCC, Manassas, VA) [34]. A LNSC line, HK [35], which resembles mesenteric LNSC functionally [16, 24, 36], was used. HK cells show the LNSC phenotype expressing CD320, CD44, α-SMA as well as CXCL12, IL-6, and IL-8 [16].

UM-UC-3 cells were cultured in Dulbecco's Modified Eagle's Medium (DMEM) with 10% fetal bovine serum (FBS, Life Technologies, Grand Island, NY), 2 mM glutamine, 100 U/ml penicillin G, and 100 mg/ml streptomycin (Irvine Scientific, Santa Ana, CA) at 37° C in a 5% CO_2_ humidified incubator. HK cells were maintained in RPMI media supplemented with 10% FBS, 2 mM glutamine, 100 U/ml penicillin G, and 100 mg/ml streptomycin [37]. HK cells can be isolated, grown, and expanded for up to 15 passages *in vitro*.

### Animals

All animal studies were conducted under approved guidelines of the Institutional Animal Care and Use Committee (IACUC) of Ochsner Clinic Foundation and in accordance with Animal Research guidelines. NOD/SCID mice were purchased from the Jackson Laboratory (Bar Harbor, ME), and acclimated for at least 10 days before being used for experiments at 6–8 weeks old.

### Patient specimens

The UCC tumors from consented patient 15 (BlCaPt15, pT3b N1 M0) and 37 (BlCaPt37, pT3b pN0 M0) were collected at resection surgery in accordance with the Ochsner Clinic Foundation Investigative Review Board (IRB) and the ethical standards of the Institutional Committee on Human Experimentation. Histological diagnoses were based on microscopic features of carcinoma cells, the histological type and grade determined by a board-certified genitourinary pathologist (Table 2).

### Tagging UCC cells with luciferase and red fluorescent protein (Luc/RFP)

UM-UC-3 cells were transduced using premade firefly luciferase /RFP expressing lentiviral vector particles according to the manufacturer's instructions (LVP402, GenTarget Inc., San Diego, CA). Briefly, UM-UC-3 cells were cultured in 500 μl DMEM with 10% FBS without antibiotics in 12-well plates (5 × 10^5^ cell/well). Luc/RFP expressing lentivirus pLentiLox3.7 (200 μl), media (300 μl), and 6 μg/ml polybrene were added to each well. After a 12 hr incubation, 2 ml of fresh media was added. RFP^+^ UM-UC-3-Luc cells were sorted using a fluorescence-activated FACS Aria cell sorter (BD, Franklin Lakes, New Jersey) repeatedly until cell RFP positivity reached more than 98% [36].

For tagging patient-derived tumor cells, the NL(CMV)Luc2-Turbo RFP/CMV/WPREDU3 lentiviral vector encoding firefly luciferase (Fluc) and TurboRFP was used. The pNL(CMV)Luc2-TurboRFP/CMV/WPREDU3 lentiviral vector plasmid encoding Fluc and TurboRFP was generated by Gateway recombination between the pENTR2B/Luc2-TurboRFP entry plasmid and the pNL(CMV)DEST/CMV/WPREDU3 destination vector [38]. The Luc2-TurboRFP sequence is composed of the Fluc coding region which was derived from the pGL4.20-Luc2/Puro plasmid (Promega, Madison, WI), and the TurboRFP coding region was derived from the pTurboRFP-N vector (evrogen; Axxora, LLC Farmingdale, NY). A spacer sequence was used to connect the Fluc and TurboRFP open reading frames. The spacer sequence was prepared by GenScript USA (Piscataway, NJ). It encodes a furin cleavage site (RAKR), a V5 epitope (GKPIPNPLLGLDST), a linker (SGSG), and the foot-and mouth-disease derived F2A peptide (APVKQTLNFDLLKLAGDVESNPGP) [39]. Lentiviral vectors were prepared as described before [40, 41]. When xenografts grew to around 1 cm in diameter as described below, tNL(CMV)Luc2-TurboRFP/CMV/WPREDU3 expressing lentiviral vector particles were injected intra-tumorally to tag the tumor cells. Luc2-TurboRFP positive tumors were monitored by BLI and enriched by implanting more highly expressing “hot spot” pieces with the highest BLI into additional mice. Xenograft tissues were harvested at 4–6 weeks, and a single cell suspension was prepared for engraftment into NOD/SCID mice. Cancer cells were used for the PDOX models when TurboRFP positivity was above 80% as determined by FACS analysis.

### Expansion of patient specimens in xenograft and enzymatic digestion

Freshly resected human UCC specimens (BlCas) were obtained via RC in cold sterile McCoy's medium containing penicillin G (500 U/ml) and streptomycin (500 mg/ml). Tissues were mechanically minced into small pieces. Small pieces of tumor (2–3 mm^3^) were first implanted into NOD/SCID mice subcutaneously in the flank, *n* = 4 per patient, using bone marrow aspiration biopsy needles for expansion.

Enzymatic digestion of tumor specimens was performed using collagenase IV (1.5 mg/ml), hyaluronidase (20 mg/ml), and Deoxyribonuclease I (DNase I, 0.1 mg/ml, all from Sigma Aldrich, St Louis, MO) in HBSS for 2 to 3 hr at 37° C [16, 36] for cancer cell isolation. Cells passed through a 40 μm cell strainer (Corning Inc., Corning, NY) and followed by IB instillation. Varying doses of UCC cells were IB instilled into NOD/SCID female mice with a catheter as described below, in the presence or absence of LN stromal HK cells with the aid of an Olympus dissecting microscope (up to ×100 magnification; Tokyo, Japan).

### Metastatic PDOX model

Enzymatic digestion of tumor specimens was performed [16, 36] to obtain single cell suspension of tumor cells. For IB installation, female mice were anesthetized with isoflurane (Isothesia^®^, 2.5% in 100% oxygen, 1 L/min). Once under appropriate sedation, the mice were placed in supine position and the bare back grounded to a Sure fit^®^ (Conmed corporation, Utica, NY) dispersive electrode. The mouse urethra was prepped with betadine solution. A 24-gauge Optiva^®^ angiocatheter (Smiths Medical International Ltd, Rossendale, Lancashire, UK) was used to catheterize the bladder under sterile conditions (Figure 1Aa). A 0.025” fixed core straight guide wire (Cook Medical, Bloomington, IN) was advanced one millimeter past the end of the angiocatheter (Figure 1Ab), and using a monopolar Bovie electrocautery, a current of four watts was applied to the guide wire for one second allowing for electrical irritation of the bladder mucosa. The wire was removed leaving the angiocatheter in place. A volume of 50 μl containing the predetermined concentrations of UCC cells with or without LN stromal HK cells was then injected (Figure 1Ac). Mice were observed for 1 hr after injection until fully recovered from anesthesia [31–33].

Tumor growth and metastatic burden were monitored using the IVIS Lumina Imaging System (PerkinElmer, Waltham, MA) for luciferase activity by BLI. Mice were injected intraperitoneally (IP) with 15 mg/ml luciferin (Cayman Chemical Co., Ann Arbor, MI) at 10 μl/g body weight and anesthetized using isoflurane for all imaging procedures. Images were analyzed using Living Image software (Caliper Life Sciences, Hopkinton, MA, Figure 1B). Mice were sacrificed when the luminescence efficiency at the primary tumor site reached various Photon levels pre-determined for each tumor cells. At necropsy, primary tumors and mouse lungs were harvested for BLI and weighed to evaluate primary tumor growth and distant organ metastasis. Tumors and mouse organs were fixed in formalin to quantify tumor formation and metastasis by H&E and IHC staining, and digital analysis.

### Antibodies and reagents for immunohistochemistry

Rabbit antibodies (Abs) against human Ki67 (Ki67, Thermo Fisher Scientific, Inc, Waltham, MA), human CD44 (CD44, Acris Antibodies, Inc, Rockville, MD), mouse CD31 (CD31, Abcam, Inc, Cambridge, MA), or alpha smooth muscle actin (specific to human and mouse, α-SMA, Acris Antibodies, Inc, Rockville, MD), and biotinylated anti-rabbit Ig secondary Abs (Vector Laboratories, Burlingame, CA) were used for IHC staining. Reagents used were avidin-biotin-peroxidase complexes (Vector Laboratories) and Sigma FAST 3,3-diaminobenzidine tablets (Sigma-Aldrich).

### Histological and immunohistochemical analysis

For histological evaluation, a portion of each UCC patient specimen, xenograft tumor tissue, and mouse lungs were fixed in 10% buffered formalin for paraffin embedding. Paraffin-embedded tissue was sectioned at 5-μm thickness and mounted onto Superfrost Plus^®^ slides (StatLab Medical Products, Pompano Beach, FL). Sections were used for H&E and IHC staining. For IHC staining, tissue slides were deparaffinized, rehydrated, and blocked for endogenous peroxidase activity. After high-temperature antigen retrieval in 0.01 M sodium citrate solution (pH 6.0), slides were serum-blocked, and immunostained using pre-determined concentrations of primary antibodies (α-SMA, 1 to 300; Ki67, 1 to 200; CD44, 1 to 75; and CD31, 1 to 200) overnight at 4° C. After rinsing the sections in phosphate-buffered saline (PBS), the slides were incubated for 1 hr at room temperature with biotinylated secondary Abs and followed by incubation with avidin-biotin-peroxidase complexes for 1 hr at room temperature according to the manufacturer's instructions (Vector Laboratories). The sections were developed with a 3,3′-diaminobenzidine substrate (Sigma-Aldrich), counterstained with hematoxylin and coverslipped with Permount (Fisher). All images were captured by a deconvoluting microscope using SlideBook 6.0 software (Intelligent Imaging Innovations, Inc, Denver, CO).

### Digital analysis

Paraffin embedded tumor slides were IHC stained using an anti-mouse CD31 antibody. At least 20 fields were digitally photographed using SlideBook software at 200 × magnification. Digital analysis was done using Aperio Image software (Image Scope, Vista, CA). Negative and positive (brown color) staining was selected, and the software quantitatively analyzed the percentages of positive areas in each field for statistical analysis.

### Statistical analysis

Data presented are representative of at least two similar independent experiments. To assess for statistically significant differences, unpaired *t* test and χ^2^ test (Prism Version 7; GraphPad Software, Inc, La Jolla, CA) were used. Results were considered statistically significant as *P <* 0.05, asterisks represent significance: ^*^P < 0.05; ^**^P < 0.01; and ^***^P < 0.001.

## Abbreviations

α-SMA: alpha-smooth muscle actin
BLI: Bioluminescence imaging
CD: Cluster of differentiation
H&E staining: Hematoxylin and eosin staining
HK cell: Lymph node stromal cell
IB: intra-vesical
IHC staining: Immunohistochemical staining
LN: Lymph node
LNSC: Lymph node stromal cell
Luc/RFP: Luciferase and red fluorescent protein
MIUCC: Muscle invasive urothelial cell carcinoma
PDOX model: Patient-derived orthotopic xenograft model
RC: Radical cystectomy
UCC: Urothelial cell carcinoma.
